# CT radiomics identifying non‐responders to neoadjuvant chemoradiotherapy among patients with locally advanced rectal cancer

**DOI:** 10.1002/cam4.5086

**Published:** 2022-08-01

**Authors:** Zinan Zhang, Xiaoping Yi, Qian Pei, Yan Fu, Bin Li, Haipeng Liu, Zaide Han, Changyong Chen, Peipei Pang, Huashan Lin, Guanghui Gong, Hongling Yin, Hongyan Zai, Bihong T. Chen

**Affiliations:** ^1^ Department of Radiology (Xiangya Hospital) Central South University Changsha Hunan P.R. China; ^2^ Department of Gastroenterology (The Third Xiangya Hospital) Central South University Changsha Hunan P.R. China; ^3^ National Engineering Research Center of Personalized Diagnostic and Therapeutic Technology Xiangya Hospital Changsha Hunan P.R. China; ^4^ National Clinical Research Center for Geriatric Disorders (Xiangya Hospital) Central South University Changsha Hunan P.R. China; ^5^ Hunan Key Laboratory of Skin Cancer and Psoriasis Changsha Hunan P.R. China; ^6^ Hunan Engineering Research Center of Skin Health and Disease Changsha Hunan P.R. China; ^7^ Department of General Surgery (Xiangya Hospital) Central South University Changsha Hunan P.R. China; ^8^ Department of Oncology (Xiangya Hospital) Central South University Changsha Hunan P.R. China; ^9^ Department of Pharmaceuticals and Diagnosis GE Healthcare Changsha P.R. China; ^10^ Department of Pathology, Xiangya Hospital Central South University Changsha Hunan P.R. China; ^11^ Department of Diagnostic Radiology City of Hope National Medical Center Duarte California USA

**Keywords:** CT radiomics, locally advanced colorectal cancer, neoadjuvant chemoradiotherapy, prediction, radiotherapy planning, treatment response

## Abstract

**Background and Purpose:**

Early detection of non‐response to neoadjuvant chemoradiotherapy (nCRT) for locally advanced colorectal cancer (LARC) remains challenging. We aimed to assess whether pretreatment radiotherapy planning computed tomography (CT) radiomics could distinguish the patients with no response or no downstaging after nCRT from those with response and downstaging after nCRT.

**Materials and Methods:**

Patients with LARC who were treated with nCRT were retrospectively enrolled between March 2009 and March 2019. Traditional radiological characteristics were analyzed by visual inspection and radiomic features were analyzed through computational methods from the pretreatment radiotherapy planning CT images. Differentiation models were constructed using radiomic methods and clinicopathological characteristics for predicting non‐response to nCRT. Model performance was assessed for classification efficiency, calibration, discrimination, and clinical application.

**Results:**

This study enrolled a total of 215 patients, including 151 patients in the training cohort (50 non‐responders and 101 responders) and 64 patients in the validation cohort (21 non‐responders and 43 responders). For predicting non‐response, the model constructed with an ensemble machine learning method had higher performance with area under the curve (AUC) values of 0.92 and 0.89 as compared to the model constructed with the logistic regression method (AUC: 0.72 and 0.71 for the training and validation cohorts, respectively). Both decision curve and calibration curve analyses confirmed that the ensemble machine learning model had higher prediction performance.

**Conclusion:**

Pretreatment CT radiomics achieved satisfying performance in predicting non‐response to nCRT and could be helpful to assist in treatment planning for patients with LARC.

## Graphical abstract

Non‐invasive methods to predict the response to neoadjuvant chemoradiotherapy in patients with locally advanced rectal cancer could help clinicians develop individualized treatment strategies. In this work, we retrospectively enrolled 215 patients with locally advanced rectal cancer who received neoadjuvant chemoradiation therapy, and we analyzed their pretreatment radiotherapy planning CT images for both radiomic and traditional radiological features. A machine learning model built with the selected radiomics and clinicopathological features. achieved satisfying performance for predicting non‐response to neoadjuvant chemoradiation therapy with an area under the curve (AUC) value of 0.92 for the training cohort and 0.89 for the validation cohort. The machine learning model outperformed a traditional logistic regression model. These findings support the use of radiomics for clinical decision‐making in locally advanced rectal cancer.

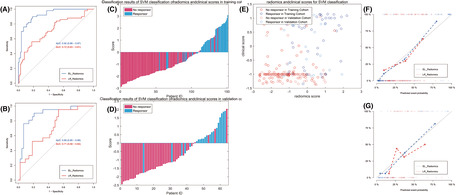

## 1 INTRODUCTION

The current standard of care for patients with locally advanced rectal cancer (LARC), especially those with advanced disease, includes neoadjuvant chemoradiotherapy (nCRT) followed by total mesorectal excision.^1^, ^2^ However, the effects of nCRT for LARC vary widely.^3^, ^4^ Although most patients with LARC experience partial response or even pathological complete response after nCRT, approximately 7% of patients with LARC may have stable disease or even disease progression and are considered non‐responders.^5^ Non‐responders fail to benefit from nCRT treatment and suffer from the side effects associated with treatment and disease progression. In addition, these patients may lose the opportunity for timely treatment with more effective therapy. Therefore, there is a need for non‐invasive methods to identify nCRT non‐responders prior to treatment and offer them a more effective treatment strategy.

Previous studies have identified various risk factors for non‐response to nCRT, including age, carcinoembryonic antigen levels, and tumor location.^6^, ^7^ These factors have been helpful in evaluating treatment response. However, they are insufficient for predicting treatment response to nCRT at an individual level in patients with LARC. Other risk factors, such as tumor heterogeneity,^8^ are important indicators of tumor resistance to nCRT and could be useful for assessing treatment response.^9^ Computed tomography (CT) is the primary imaging modality for the evaluation of tumor heterogeneity. Heterogeneity is assessed by variations in CT density and enhancement characteristics within the tumor.^10^, ^11^, ^12^, ^13^ Patients with LARC routinely undergo a pretreatment CT scan for radiotherapy planning. However, traditional radiological evaluation of CT images through visual inspection provides limited information for predicting response and additional imaging‐based strategies are needed.

Radiomics is a method for extracting high‐dimensional imaging features from medically acquired images for clinical practice, which can be used to quantitatively evaluate tumor phenotypes using computational algorithms.^14^ Radiomic features are different from traditional radiological characteristics assessed visually by radiologists. For instance, radiomics may generate advanced imaging features not visible to a human eye, including texture, wavelet, and histogram features. The extraction and identification of radiomic features rely on computational methods that are usually beyond the limits of human visual perception.^14^ The radiomic approach has advantages. First, it is non‐invasive and can be performed repeatedly on the imaging data. Second, it can provide a comprehensive review of the entire tumor. Third, it is cost‐effective since the imaging data are already acquired for clinical care. Therefore, radiomics has great potential for assisting tumor staging, treatment response assessment, and prognosis. Prior studies have identified the link between the radiomic features assessed by computational methods and tumor treatment response, which has shown the potential for imaging‐based predictive modeling.^15^, ^16^, ^17^, ^18^

Radiomics has been studied as a predictor of the response to nCRT in patients with LARC.^19^ However, most of the previous studies used radiomics to evaluate pathological complete response, and only a few studies have attempted the prediction of non‐response to nCRT in patients with LARC.^5^, ^17^, ^20^, ^21^ Among these, two studies used magnetic resonance imaging (MRI) rather than CT.^5^, ^17^ Since CT is faster, less expensive, and produces more detailed images of bowel pathology than MRI, most clinical practices acquire CT images for radiotherapy treatment planning for LARC. Therefore, it is important to thoroughly assess the value of CT radiomics for predicting treatment response.

Only two reports have evaluated the prognostic value using radiomic texture features from pretreatment CT scans for predicting response to nCRT in patients with LARC.^20^, ^21^ However, one report did not describe the differentiation performance of the model.^20^ The other study used tumor node metastasis (TNM) staging rather than the standard American Joint Committee on Cancer (AJCC) tumor regression grade (TRG) system^22^ to assess response.^21^ In addition, the unbalanced differentiation performance of their models (area under the curve [AUC]: 0.90 and 0.70 for the training and validation cohorts, respectively) implied that their results might not easily be generalizable. Therefore, more work needs to be done to understand the potential of CT‐based radiomics models to predict nCRT response.

In this study, we conducted a retrospective review of 215 patients who received standard nCRT before surgery and who had subsequent pathological confirmation of LARC on the surgical specimens. We used machine learning methods to develop radiomic models based on the radiotherapy treatment‐planning CT images for prediction of non‐response, and we also assessed the model performance in clinical application analysis.

## 2 MATERIALS AND METHODS

### 2.1 Patients

This study was approved by the institutional review board of Xiangya Hospital at Central South University, P.R. China. (IRB # 201910070). The board waived the requirement for written informed consent from patients due to the retrospective nature of this study.

Consecutive patients who had pathologically confirmed LARC (cT3/T4N_x_M_0_) on surgical specimens and who had received nCRT and total mesorectal excision from March 2009 to March 2020 at our hospital, were included in this study. Specifically, we included patients classified as cT3/T4N_x_M0 using the TNM system, because patients with this stage of disease were recommended to receive nCRT. The cT3/T4N_x_M_0_ staging indicates T3 or T4 disease, with or without lymph node involvement and without metastases.

The nCRT regimen was carried out in the following procedures. Patients received 46–50 Gy total dose of radiation therapy delivered in 23–25 fractions 5 days per week for the duration of radiation plus concomitant 5‐Fu‐based chemotherapy. The radiation clinical target volume included the primary rectal cancer, perirectal and internal iliac nodes, mesorectum, pelvic sidewalls, and presacral space with the upper border at the sacral promontory. The concurrent chemotherapy regimens included mono‐chemotherapy of 5‐FU or the combined chemotherapy of mFOLFOX6, 5‐FU, leucovorin and oxaliplatin or capecitabine. In our study, all included patients completed nCRT treatment as planned and those who discontinued for some reason or had a dose reduction due to toxicity were not included in the final cohort.

**FIGURE 1:**
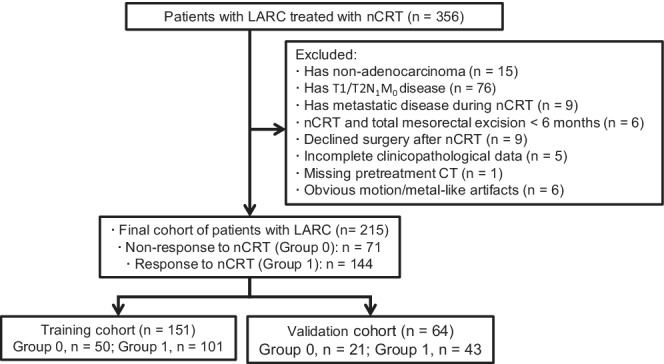
Flow chart of the enrollment process for patients with LARC who received nCRT. CT, computed tomography; LARC, locally advanced rectal cancer; nCRT, neoadjuvant chemoradiotherapy.

The enrollment of this study cohort, including inclusion and exclusion, is presented in Figure 1.

### 2.2 Pathology re‐assessment

Two experienced pathologists (H. Y. and G. G., with 25 and 7 years of experience in gastrointestinal cancer, respectively) independently re‐analyzed the pathological specimens for the enrolled patients. Additional sections were obtained from paraffin blocks when needed. The two pathologists were blinded to the pathological results as well as the CT and clinical data. Pathological regression of the tumors was judged according to the TRG system from AJCC.^22^ Patients with TRG 3 (minimal evidence of tumor response) were categorized as non‐responders, and patients with TRG 0–2 (TRG 0, no residual tumor cells; TRG 1, single tumor cell or small group of tumor cells; TRG 2, residual cancer with desmoplastic response) were categorized as the responder group. The findings were recorded once a consensus was reached between the pathologists.

### 2.3 CT image acquisition and analysis

All CT scans were conducted using a standard protocol in the same 16‐detector row spiral CT (Brilliance 16, Philipps) scanner. The enhanced CT images were obtained after the patients received a 90–100 ml intravenous bolus injection of a non‐ionic iodinated contrast agent (iopromide; Ultravist; Schering AG) at a rate of 3–3.5 m/s. The axial CT scans were accessed from our Picture Archiving and Communication System (PACS, Carestream), and all the original CT images underwent 256‐bin normalization using a gray‐scale discretization method prior to extraction of the radiomic features (Analysis Kit software, version V3.0.0.R, GE Healthcare). An axial thickness of 3 mm was used to reconstruct all the CT images.

Independent review of the CT images was conducted by two radiologists who specialize in body imaging (Changyong Chen and Haipeng Liu, with 25 and 7 years of experience, respectively). Disagreements between Changyong Chen and Haipeng Liu were resolved by one additional senior radiologist (Zaide Han, over 35 years of experience). The radiologists reached consensus through discussion if there were discrepancies. For each tumor, traditional radiological characteristics (derived from visual inspection) were assessed, including the tumor location, boundary, size, distance from the lower edge of the tumor to the anal canal, and the TNM stage (based on CT imaging).

### 2.4 Image segmentation and texture feature extraction

The enhanced CT images at the largest tumor diameter were selected for all patients and the tumor margins were carefully contoured on the selected images. Subsequently, the tumors were segmented to generate quantitative features by texture analysis (MaZda Version 4.6, Technical University of Lodz). Caution was taken during tumor segmentation to minimize interference with the adjacent normal tissues, intestinal contents, air and fat outside the tumor. For each tumor, 275 quantitative features were extracted, including a gray‐level histogram, gradient, run‐length matrix, co‐occurrence matrix, autoregressive model, and wavelet transform analysis.

### 2.5 Reproducibility of radiomic feature extraction

In this study, intraclass correlation coefficient (ICC) was used to access the inter‐ and intra‐observer reproducibility.^19^, ^20^ Specifically, a total of 50 patients were randomly selected and their CT images were evaluated by two radiologists (Reader 1 and Reader 2) to access the inter‐observer reproducibility. They evaluated the 50 CT images dependently. In addition, to assess the intraobserver reproducibility, Reader 1 generated texture features twice with the same procedure over an 8‐week interval. The ICC for both Reader 1 (first attempt at feature extraction) and Reader 2 reached the highest at 0.846 and the lowest at 0.762. The intra‐observer ICC ranged from 0.788 to 0.872 for Reader 1 through the two attempts at radiomic feature extraction. Generally, an ICC >0.75 was indicative of good agreement. In addition, a third radiologist (X. Y.) was brought in to assess the tumor segmentation and feature extraction in the event of discrepancy between these two readers. Consensus was reached through discussion.

### 2.6 Statistical analysis, feature selection, and predictive modeling

All statistical analyses were performed with IBM SPSS version 20.0.0 (IBM Corporation). To evaluate the differences between groups for the quantitative features, Student's *t* test (normally distributed data) and the Wilcoxon rank‐sum test (non‐normally distributed data) were used. For the qualitative features, a chi‐square test or Fisher's exact test was used. MATLAB 2017a (Mathworks, Inc.) was used for data processing, feature selection, and model building.

Two machine learning models were built using two different methods, that is, the ensemble learning (EL) method and the logistic regression method (LR model). The EL model was constructed using the training cohort and confirmed using the validation cohort.^19^ Specifically, after extracting the textures from the pretreatment CT through Mazda, the least absolute shrinkage and selection operator (LASSO) method was used to select the most useful predictive features. Besides, radiomic score (Rad‐score) was also calculated for each patient as a linear combination of selected features that were weighted by their respective coefficients. Based on these selected features, a classification model was constructed using random forest (RF) method, and the RF score was generated. Based on the results of the LASSO and the RF‐score, the support vector machine (SVM) method (SVMscore) was used to build the combined classification model (SVM1). In addition, similar procedures were undertaken on the CT imaging data obtained through traditional radiological assessment and clinicopathological data to build a second SVM model, that is., the SVM2 model. Subsequently, the SVM1 and SVM2 were integrated with the EL algorithm to get the final SVM model, that is, the EL model (Figure S1). The hyperparameters of the models were determined by the LASSO method. In addition, the models constructed with logistic regression method (LR model) were also built and examined.^20^

**FIGURE 2:**
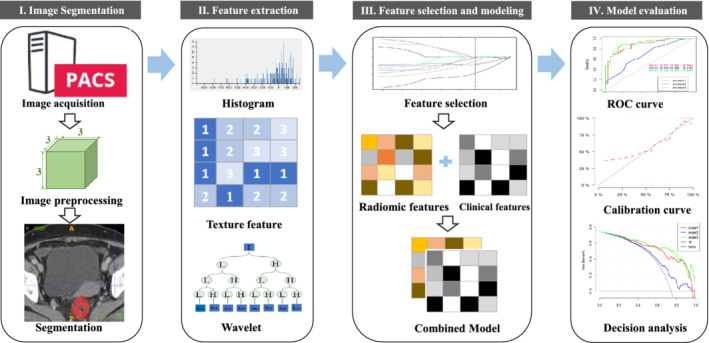
Workflow for the present study showing tumor segmentation on the computed tomography images, radiomic feature extraction and selection, and predictive modeling. PACS, picture archiving and communication system; ROC, receiver operating characteristic.

Calibration of the model was evaluated using the Hosmer‐Lemeshow H test, and receiver operating characteristic curves were used to assess the differentiation efficiency of the training and validation cohorts. Decision curve analysis was used for the validation cohort to evaluate the clinical usefulness of the model. To evaluate the correlations among all model features, correlation matrix analysis was performed. A *p* < 0.05 was considered statistically significant. Figure 2 presents the workflow for tumor segmentation, feature extraction, and predictive modeling.

## 3 RESULTS

### 3.1 Cohort characteristics

A total of 215 patients, consisting of 71 non‐responders and 144 responders, were included in the analysis (Table 1). The differences between the groups were statistically significant for age (*p* = 0.012) and platelet count (*p* = 0.005). Patients were randomized into the training cohort and validation cohort in a 2:1 ratio. No differences were observed between the training and validation cohorts in this model.

**TABLE 1:** Demographic, clinicopathological, and laboratory data for the 215 patients with locally advanced rectal cancer who received nCRT

	No‐response (*N* = 71)	Response (*N* = 144)	*p* value	Training cohort (*N* = 151)	Validation cohort (*N* = 64)	*p* value
Demographics and clinical characteristics						
Sex						
Male	47 (66.20%)	84 (58.33%)	0.173	93 (61.59%)	38 (59.38%)	0.761
Female	21 (29.58%)	60 (41.67%)		58 (38.41%)	26 (40.62%)	
Age	49.54 ± 12.63	53.84 ± 9.02	0.012*	52.88 ± 10.35	51.33 ± 10.92	0.324
Imaging characteristics						
Clinical T stage (pretreatment)						
T3	51 (30.7%)	115 (69.3%)	0.187	115 (76.2%)	51 (79.7%)	0.573
T4	20 (40.8%)	25 (17.36%)		36 (23.8%)	13 (20.3%)	
Clinical N stage (pretreatment)						
N−	9 (12.7%)	27 (18.8%)	0.262	23 (15.2%)	13 (20.3%)	0.362
N+	62 (87.3%)	117 (81.3%)		128 (84.6%)	51 (79.7%)	
CT Value	56.44 ± 12.59	57.86 ± 11.41	0.410	57.90 ± 12.32	56.20 ± 10.46	0.304
Distance from mass to anal canal	5.13 ± 1.99	5.00 ± 1.91	0.647	4.88 ± 1.91	5.44 ± 1.95	0.051
Laboratory findings						
Red cell count, ×10^9^ per L	4.53 ± 0.59	4.48 ± 0.57	0.588	4.53 ± 0.58	4.42 ± 0.55	0.167
HGB	133.41 ± 20.74	129.14 ± 21.66	0.170	131.72 ± 20.90	127.78 ± 22.49	0.218
PLT, ×10^9^ per L	226.06 ± 65.06	258.03 ± 82.45	0.005*	245.90 ± 79.27	251.19 ± 76.95	0.652
NEU, ×10^9^ per L	3.86 ± 1.44	4.09 ± 1.49	0.282	4.11 ± 1.51	3.81 ± 1.37	0.181
Lymphocyte count, ×10^9^ per L	1.65 ± 0.64	1.73 ± 0.58	0.385	1.73 ± 0.61	1.63 ± 0.56	0.274
Monocyte, ×10^9^ per L	0.45 ± 0.16	0.46 ± 0.16	0.516	0.46 ± 0.17	0.44 ± 0.14	0.299
NLR	2.68 ± 1.43	2.64 ± 1.60	0.869	2.70 ± 1.68	2.57 ± 1.18	0.583
LMR	4.00 ± 1.89	4.04 ± 1.54	0.870	4.06 ± 1.70	3.93 ± 1.56	0.594
PLR	157.44 ± 71.38	164.10 ± 69.85	0.515	158.49 ± 70.51	169.94 ± 69.57	0.276
RDW	13.68 ± 2.15	13.76 ± 1.99	0.781	13.54 ± 1.43	14.19 ± 3.00	0.103
Mean platelet volume	9.97 ± 1.51	9.70 ± 1.72	0.262	9.89 ± 1.72	9.56 ± 1.50	0.191
Albumin (g/L)	43.15 ± 4.59	43.04 ± 4.44	0.870	43.25 ± 4.46	42.67 ± 4.52	0.388
Globulin (g/L)	27.77 ± 4.01	28.02 ± 4.73	0.699	27.72 ± 4.60	28.44 ± 4.24	0.283
Albumin/globulin ratio	1.58 ± 0.29	1.58 ± 0.30	0.939	1.60 ± 0.29	1.53 ± 0.28	0.105
Blood sugar (mmol/L)	5.45 ± 1.05	5.44 ± 1.07	0.951	5.34 ± 0.78	5.68 ± 1.52	0.091
BUN (mmol/L)	4.69 ± 1.51	4.57 ± 1.33	0.555	4.62 ± 1.33	4.60 ± 1.53	0.941
Scr (μmoI/L)	88.04 ± 23.96	83.42 ± 17.00	0.105	86.17 ± 20.73	82.05 ± 16.57	0.159
TG (mmol/L)	1.64 ± 1.11	1.61 ± 1.22	0.854	1.68 ± 1.08	1.49 ± 1.40	0.299
Cho (mmol/L)	4.71 ± 0.99	4.85 ± 0.94	0.342	4.82 ± 0.97	4.76 ± 0.93	0.692
HDL (mmol/L)	1.27 ± 0.39	1.26 ± 0.28	0.829	1.26 ± 0.34	1.27 ± 0.29	0.840
LDL (mmol/L)	2.89 ± 0.73	2.98 ± 0.73	0.389	2.97 ± 0.77	2.89 ± 0.64	0.422
Fecal occult blood test						
Positive	11 (15.50%)	26 (18.06%)	0.704	121 (80.13%)	57 (89.06%)	0.113
Negative	60 (84.51%)	118 (81.94%)		30 (19.87%)	7 (10.94%)	
CEA (ng/ml)	11.07 ± 29.07	14.47 ± 25.82	0.384	12.86 ± 26.28	14.49 ± 28.54	0.685
CA199 (U/ml)	29.06 ± 68.84	26.33 ± 71.45	0.790	23.63 ± 66.89	35.73 ± 78.12	0.251
CA125 (U/ml)	12.74 ± 39.85	10.60 ± 31.23	0.668	9.87 ± 29.78	14.69 ± 43.06	0.347
Pathology findings						
Pathology type						
Well‐differentiated adenocarcinoma	64 (90.15%)	135 (93.75%)	0.524	140 (92.72%)	59 (92.19%)	1
Poorly differentiated adenocarcinoma	4 (5.63%)	6 (4.17%)		7 (4.63%)	3 (4.69%)	
Mucinous carcinoma and mixed carcinoma	3 (4.22%)	3 (2.83%)		4 (2.65%)	2 (3.12%)	
Treatment response						
Response to nCRT						
Non‐response	–	–	–	50 (33.11%)	21 (32.81%)	0.966
Response	–	–		101 (66.89%)	43 (67.19%)	

Abbreviations: BUN, blood urea nitrogen; CA125, carbohydrate antigen199; CA199, carbohydrate antigen199; CEA, carcino‐embryonic antigen; Cho, cholesterol; CT, computed tomography; HDL, high‐density lipoprotein; HGB, hemoglobin; LDL, low‐density lipoprotein; LMR, lymphocyte‐to‐monocyte ratio; nCRT, neoadjuvant chemoradiotherapy; NEU, neutrophil; NLR, neutrophil‐to‐lymphocyte ratio; PLR, platelet‐to‐lymphocyte ratio; PLT, platelet; RDW, red cell distribution width; Scr, serum creatinine level; TG, triacylglycerol.

* *p* < 0.05.

### 3.2 Predictive modeling

**FIGURE 3:**
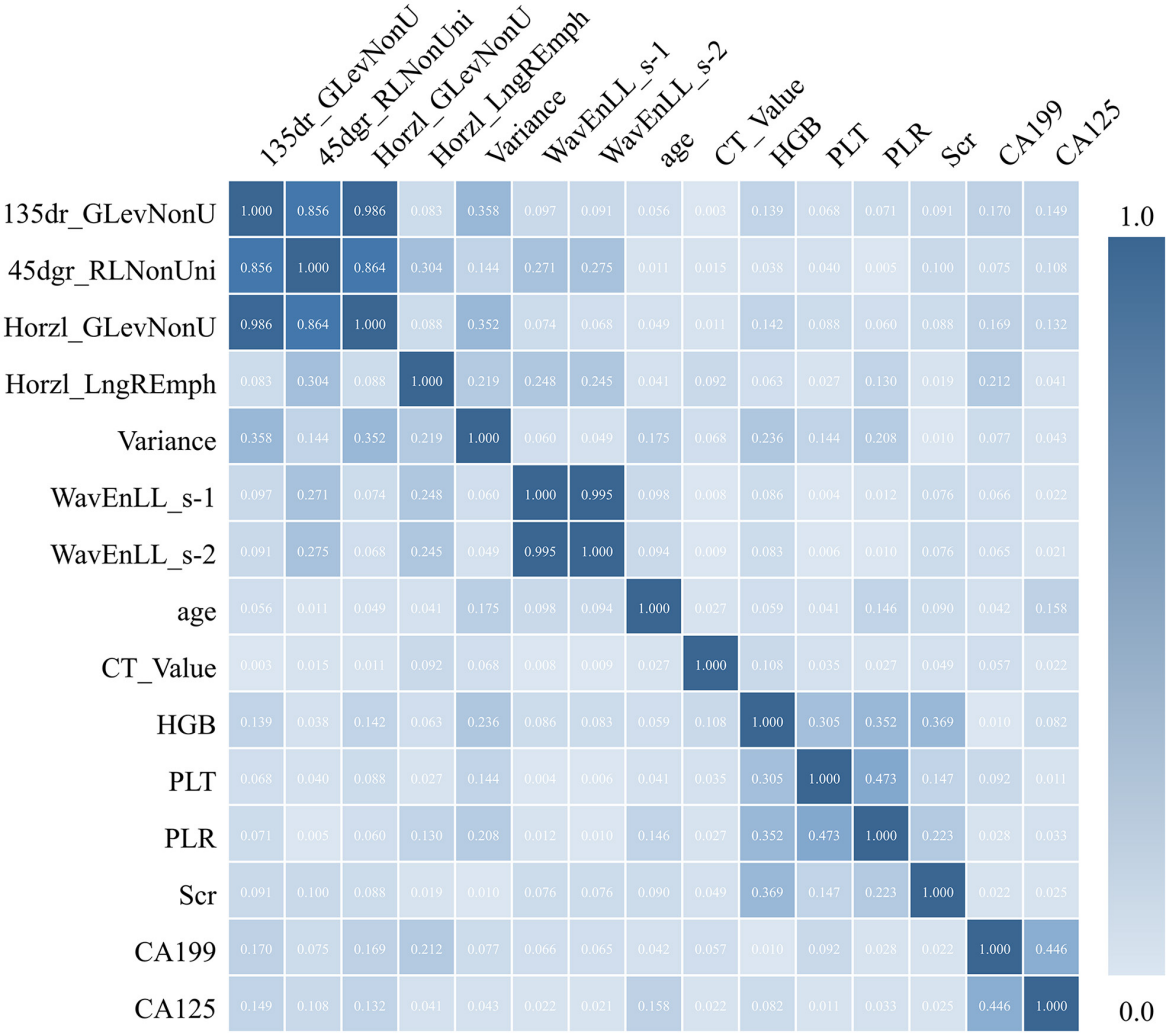
Correlation matrix testing for the 15 selected features, including the seven radiomic features and the eight clinicopathological features, for building prediction models. There were low correlations among the features, indicating their varying contributions rather than having redundant tumor information.

The EL model for predicting non‐response was constructed.^19^ In the final prediction model, a total of 15 features, including seven CT radiomic features and eight clinicopathological features, were selected by using our previously established algorithm.^17^, ^19^ Figure 3 shows the correlation matrix map for all 15 selected features. Weak correlations were observed among most features, with the largest being smaller than 0.1000. Nevertheless, strong correlations were also observed but only among a few features. For example, the correlation coefficient between 135dr_GlevNonu and Horzl_GLevNonU was 0.986. This indicates that these features may reflect different tumor characteristics and therefore may enhance the classification performance.

### 3.3 Model performance

**FIGURE 4:**
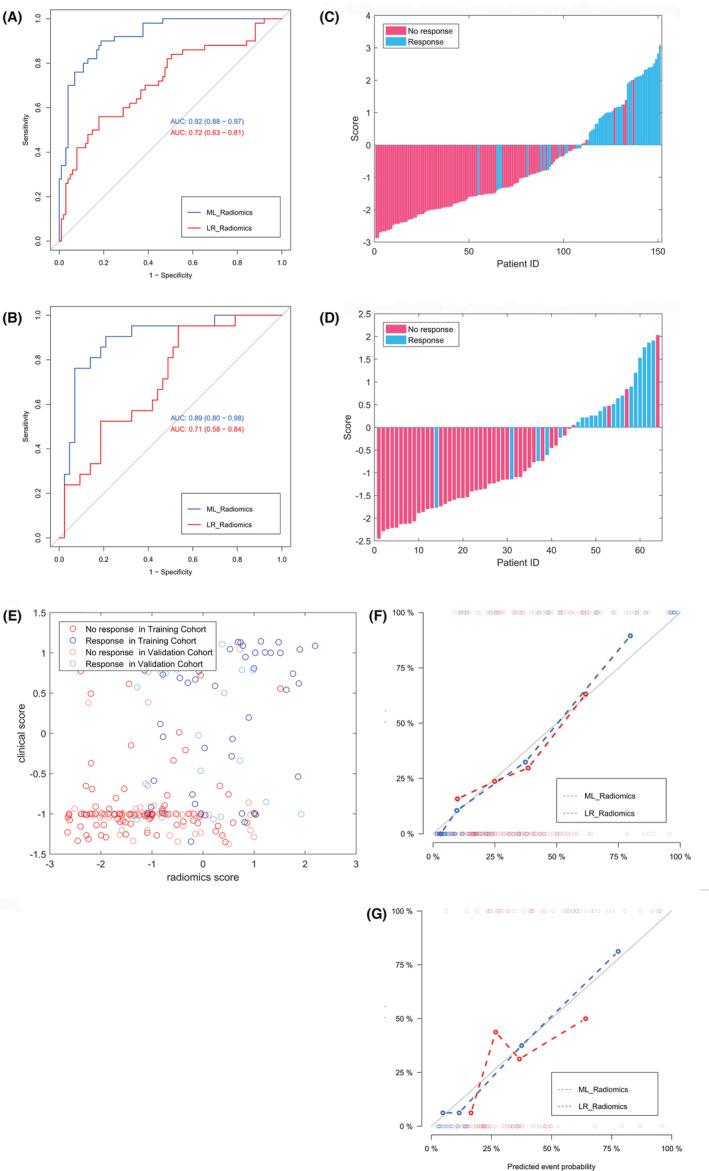
Performance of the EL radiomic model compared to the LR radiomic model. (A and B) ROC curves of the two models for the training cohort (A) and the validation cohort (B). (C and D) Classification results for the training cohort (C) and validation cohort (D). (E) Case distribution map for radiomic and clinical scores. (F and G) The calibration curves calculated for the training cohort (F) and the validation cohort (G). AUC, area under the curve; EL, ensemble‐learning; LR, logistic regression; ROC, receiver operating characteristic.

For the training cohort, the EL model yielded an AUC of 92.40% (sensitivity 88.00% and specificity 86.14%). For the validation cohort, the EL model yielded an AUC of 88.93% (sensitivity 90.48% and specificity 86.05%) (Figure 4A,B). Figure 4A–E demonstrates the good performance of the differentiation models.

The LR model was constructed in a similar fashion as previously.^18^, ^20^ The differentiation efficiency of the two models is shown in Figure 4. The LR model yielded an AUC of 72.10% in the training cohort (sensitivity = 56.00% and specificity = 82.35%), and an AUC of 71.85% in the validation cohort (sensitivity = 95.20% and specificity = 47.50%), which were both lower than those in the EL model (Figure 4A,B).

For the EL model, the calibration curve showed good agreement between prediction and observation of non‐response in both the training and validation cohorts. However, the agreement between prediction and observation was less robust for the LR model (Figure 4F,G).

### 3.4 Clinical application

**FIGURE 5:**
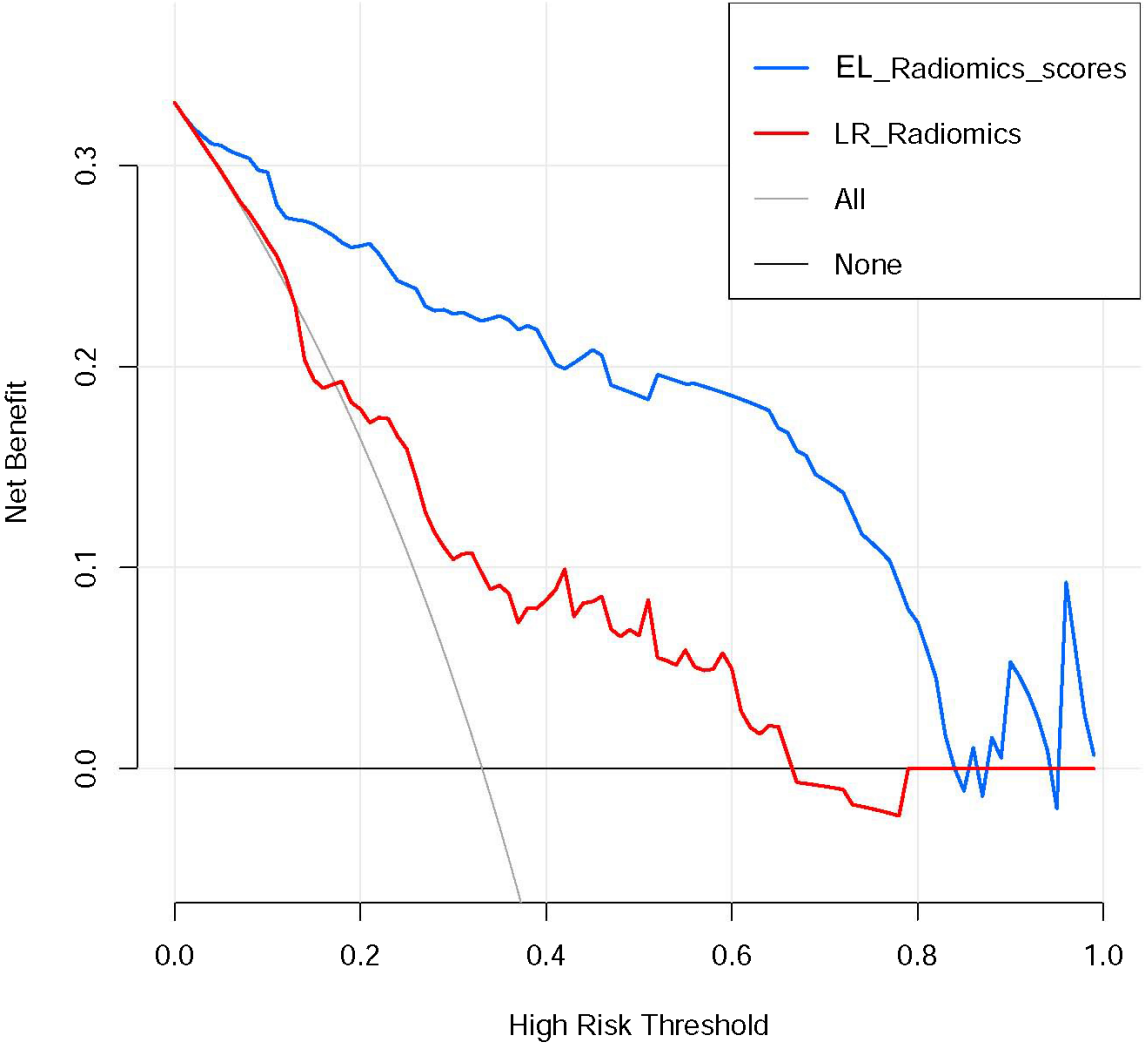
Decision curve analysis for the ensemble‐learning (EL) and logistic regression (LR) radiomic models. The horizontal gray line represents the assumption that no patients are at risk of non‐response to neoadjuvant chemoradiotherapy (nCRT) and the oblique gray line represents the assumption that all patients would develop non‐response to nCRT.

The decision curve analysis revealed that if the threshold probability of a patient or doctor was >5%, using the EL radiomic model to predict non‐response was more beneficial than using either the diagnose‐all or diagnose‐none response scheme alone (Figure 5).

## 4 DISCUSSION

In this study, we presented a pretreatment radiotherapy planning CT radiomics for the prediction of non‐response to nCRT in patients with LARC. In addition, we also showed that a prediction model built with the ensemble machine learning method performed better than a model built with the LR method.

Radiomics is useful for quantitatively assessing tumor heterogeneity, which is thought to underlie variability in responses to nCRT.^11^, ^18^, ^23^, ^24^ Rectal cancers tend to be heterogeneous due to bleeding, cystic degeneration and necrosis, and rapid proliferation, all of which contribute to resistance to chemoradiotherapy.^11^, ^25^ Our results identified several radiomic features that reflect tumor heterogeneity and were relevant to predicting response to nCRT in patients with LARC. These radiomic features included one histogram feature (Variance), four run‐length matrix features (135dr_GLevNonU, Horzl_GLevNonU, 45dgr_RLNonUni, Horzl_LngREmph), 1 co‐occurrence matrix feature (Variance), and two Wavelet parameters (WavEnLL_s‐1, WavEnLL_s‐2). It should be noted that 87.5% (5/8) of these features belonged to the run‐length and co‐occurrence matrix features, which have been categorized as texture features known to represent tumor heterogeneity in different dimensions.^14^ In addition, the remaining features, including the histogram feature (Variance), reflect the geometric characteristics of the tumors. In addition, wavelet parameters, which decompose the original images for further texture analysis, also contributed to tumor heterogeneity. Further comparative radiological‐pathological studies will be helpful for clarifying the pathological basis and clinical significance of these radiomics features. We believe the potential for improving interpretability of radiomics for the prediction of treatment response may be assessed in several ways. First, correlation analysis may be performed between radiomics and traditional radiological response criteria such as tumor size measurements, lymph node involvement, CT enhancement characteristics, etc. This approach may help to understand how radiomics may be associated with the routine imaging response features that are commonly used in clinical practice. Second, radiomic features may also be compared to metabolic imaging such as positron emission tomography (PET)/CT scans which are routinely used to gauge response to treatment. Third, radiomic features may reflect the biological behavior of the tumor such as tumor heterogeneity and aggressiveness, which should help to predict response to treatment. Lastly, we should explore how radiomics may reflect or be associated with pathological findings such as tumor neovascularity and necrosis, and tumor markers such as Carcinoembryonic Antigen (CEA), which are known to be important for the prediction of treatment response.

Our CT radiomic prediction model achieved a satisfying performance for predicting non‐response to nCRT in patients with LARC, which is consistent with the published literature.^20^, ^21^ In addition, our model had better performance than the model built by Vandendorpe et al, as evidenced by our AUC values of 0.92 and 0.89 in the training and validation cohorts respectively, which were higher than their values of 0.90 and 0.70.^21^ We speculate our improved model performance might be due to our study design. First, we enrolled over 200 patients, whereas the previous studies had lower sample sizes of 121 and 95 patients.^20^, ^21^ Second, we used routine pretreatment radiotherapy planning CT images for radiomic analysis and all the CT images for our cohort were obtained from the same scanner with the same scanning protocol, which largely avoided imaging variability although it may pose issues with generalizability of our study results. Our approach should be clinically relevant since almost all patients with LARC undergo a CT scan prior to treatment as part of standard clinical care. Third, our model included clinicopathological data and traditional radiological characteristics, including CT density values and platelet counts, in addition to radiomic features. Our approach was reasonable since these characteristics are known to be related to treatment response after nCRT.^26^ Our low correlation index between the CT density value and the other features also indicated that both the radiomic and traditional CT features contributed non‐redundant tumor information, thus improving the model performance. Lastly, in addition to LR,^20^ we also developed the algorithm using an ensemble machine learning classification method.^17^, ^19^ Our study showed that the EL model with an optimized algorithm achieved better performance than the LR model, indicating the potential of using the EL model as a non‐invasive method to predict treatment response in LARC patients.

Some of the clinical indicators identified in the present study, such as age, are relevant to treatment response as reported previously. For instance, our study identified age as a predictor of treatment response, suggesting that older patients may benefit from nCRT.^6^ Also, the platelet‐to‐lymphocyte ratio, a systemic inflammatory indicator predicting attenuated tumor immunity and poor prognosis in many cancer types, which has been associated with poor nCRT response,^27^, ^28^, ^29^ was a powerful predictor of poor response. In addition, our finding that serum creatinine as a new predictor for treatment response was not surprising. Serum creatinine reflects the renal clearance rate of chemotherapeutic agents, which is directly related to the concentration and thus the effect of the nCRT. However, we were surprised that the pre‐nCRT hemoglobin levels were negatively related to tumor response. Tumor hypoxia is a strong predictor of chemoradiotherapy resistance,^30^ and prior studies have found this parameter to be positively related to treatment response.^31^, ^32^ We speculated that the pre‐nCRT hemoglobin may be an indicator of overall oxygen capacity but not a sufficient surrogate of oxygen content in the local tumor environment. Nevertheless, the mechanism for the negative relationship between hemoglobin levels and treatment response in our study was not clear.

Our study has several limitations. First, this was a retrospective study conducted at a single center, and potential selection bias was unavoidable. This single‐center study with CT images obtained on the same scanner may limit the generalizability of our results, which needs to be tested and validated in a multicenter multi‐scanner setting. Second, our sample size was modest, which may affect the stability and accuracy of the predictive models. We did not perform deep learning analysis out of concern for our modest sample size and potential issues with unexplainable data generated in a “black box” through deep learning approach. Finally, our radiomic features were extracted on a cross‐sectional two‐dimensional plan on the CT images encompassing the largest tumor diameter, which may result in loss of tumor information during the tumor segmentation as compared to a three‐dimensional approach. Nevertheless, previous studies have demonstrated the reliability of the two‐dimensional method.

In summary, we present a non‐invasive radiomic approach that uses clinically acquired pretreatment radiotherapy planning CT images to predict nCRT response in patients with LARC. The findings from the present study indicate the potential for developing personalized treatment strategies for patients with LARC.

## AUTHOR CONTRIBUTIONS

Zinan Zhang and Xiaoping Yi analyzed the data and wrote the first draft of the manuscript. Qian Pei, Yan Fu, and Bin Li collected the data. Haipeng Liu, Zaide Han, Changyong Chen, Peipei Pang, and Huashan Lin helped with the statistical analysis. Guanghui Gong, Hongling Yin, and Hongyan Zai were major contributors in manuscript preparation. Bihong T. Chen reviewed the study design and data analysis and edited the manuscript critically. All authors read and approved the final manuscript.

## FUNDING INFORMATION

This study was funded in part by the Natural Science Foundation of Hunan Province, P. R. China (2022JJ30979), the Xiangya‐Peking University, Wei Ming Clinical and Rehabilitation Research Fund (No. xywm2015I35), and China Post‐Doctoral Science Foundation (2018M632997, 2022M713536). The funders had no role in the study design, data collection, data analysis, and interpretation, manuscript preparation; or the decision to submit the paper for publication.

## CONFLICT OF INTEREST

The authors declare no conflicts of interest.

## ETHICS STATEMENT

Institutional Review Board approval was obtained (IRB#: 201910070).

## Supporting information


Figure S1

## Data Availability

Data available on request from the authors.
