# Supplementary material for: CT radiomics identifying non‐responders to neoadjuvant chemoradiotherapy among patients with locally advanced rectal cancer
Source: Cancer Med. 2022 Aug 1;12(3):2463–73. doi: 10.1002/cam4.5086 (PMC9939108; doi:10.1002/cam4.5086)
Supplement: Supplementary file 1 — Figure S1 [file CAM4-12-2463-s001.docx]

**Supplementary material:**


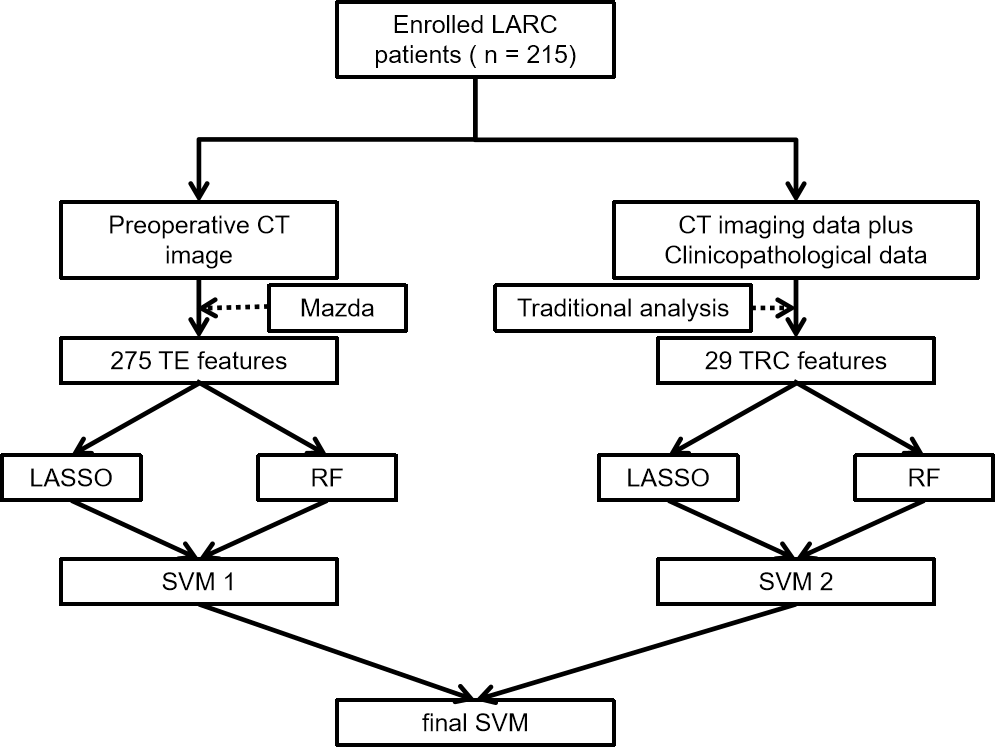


Figure 1S. The flow chart for construction of the ensemble learning (EL) model. LARC, locally advanced rectal cancer; TE, texture features; TRC, Traditional radiological features; LASSO, least absolute shrinkage and selection operator; RF, Random Forest; SVM, support vector machine.
